# Ultra High-Resolution Gene Centric Genomic Structural Analysis of a Non-Syndromic Congenital Heart Defect, Tetralogy of Fallot

**DOI:** 10.1371/journal.pone.0087472

**Published:** 2014-01-31

**Authors:** Douglas C. Bittel, Xin-Gang Zhou, Nataliya Kibiryeva, Stephanie Fiedler, James E. O’Brien, Jennifer Marshall, Shihui Yu, Hong-Yu Liu

**Affiliations:** 1 The Ward Family Heart Center, Children’s Mercy Hospitals and Clinics and University of Missouri-Kansas City School of Medicine, Kansas City, Missouri, United States of America; 2 Section of Cardiovascular Surgery, The First Affiliated Hospital of Harbin Medical University, Harbin, China; 3 Department of Pathology, Children’s Mercy Hospitals and Clinics and University of Missouri-Kansas City School of Medicine, Kansas City, Missouri, United States of America; 4 Department of Laboratory Medicine and Pathology, Seattle Children's Hospital and Department of Laboratory Medicine, University of Washington School of Medicine, Seattle, Washington, United States of America; CNRS UMR7275, France

## Abstract

Tetralogy of Fallot (TOF) is one of the most common severe congenital heart malformations. Great progress has been made in identifying key genes that regulate heart development, yet approximately 70% of TOF cases are sporadic and nonsyndromic with no known genetic cause. We created an ultra high-resolution gene centric comparative genomic hybridization (gcCGH) microarray based on 591 genes with a validated association with cardiovascular development or function. We used our gcCGH array to analyze the genomic structure of 34 infants with sporadic TOF without a deletion on chromosome 22q11.2 (n _male_ = 20; n _female_ = 14; age range of 2 to 10 months). Using our custom-made gcCGH microarray platform, we identified a total of 613 copy number variations (CNVs) ranging in size from 78 base pairs to 19.5 Mb. We identified 16 subjects with 33 CNVs that contained 13 different genes which are known to be directly associated with heart development. Additionally, there were 79 genes from the broader list of genes that were partially or completely contained in a CNV. All 34 individuals examined had at least one CNV involving these 79 genes. Furthermore, we had available whole genome exon arrays from right ventricular tissue in 13 of our subjects. We analyzed these for correlations between copy number and gene expression level. Surprisingly, we could detect only one clear association between CNVs and expression (*GSTT1*) for any of the 591 focal genes on the gcCGH array. The expression levels of *GSTT1* were correlated with copy number in all cases examined (r = 0.95, p = 0.001). We identified a large number of small CNVs in genes with varying associations with heart development. Our results illustrate the complexity of human genome structural variation and underscore the need for multifactorial assessment of potential genetic/genomic factors that contribute to congenital heart defects.

## Introduction

Tetralogy of Fallot (TOF) consists of a ventricular septal defect, obstruction of the right ventricular outflow tract, override of the ventricular septum by the aortic root, and right ventricular hypertrophy [1]. As the most prevalent form of cyanotic heart disease, it occurs in about five to seven per 10,000 live births, and accounts for 5–7% of all congenital heart defects (CHD). The occurrence of CHD in the offspring of mothers with TOF is approximately 3.1%, supporting a genetic contribution to the developmental defect [2]–[4]. With the tremendous advancements in surgical and medical management, the morbidity and mortality of those born with TOF in the last 20 years is significantly improved. However, long-term sequelae, including ventricular dysfunction, arrhythmia, and life-long disability, are still challenges in this patient population [1], [5]. As with most congenital heart defects, the etiology of TOF is poorly understood. Improved understanding of possible causes will permit insight into the pathobiological basis of TOF, and allow for improved definition of disease risk. It may also empower health care practitioners with improved diagnosis, prognosis, and therapy.

Based on studies of recurrence and transmission risks, it is hypothesized that the etiology of all CHD (including TOF) is most likely multifactorial in origin [2], [6]. The sporadic nature of most non-syndromic CHD probably involves a multitude of susceptibility genes with low-penetrance mutations (common variants), or intermediate-penetrance mutations (rare variants), superimposed on unfavorable environmental factors. Conceptually, an individual’s genetic configuration interacts with environmental and epigenetic factors to reach a threshold of “noise” in the cell to cell communication exceeding the ability of feedback regulatory mechanisms to compensate, resulting in a congenital heart defect [7]. Although widely accepted, this hypothesis remains difficult to prove, and only a few studies describing accumulation and/or interaction effects in CHD have been reported [8].

The influence of genetic factors is evident by the association of TOF with chromosomal abnormalities such as microdeletion of chromosome 22q11.2, and trisomy 21, 18, or 13 [5]; however, these patients usually have multiple non-cardiac abnormalities concurrently with TOF. Multiple genetic studies have shown many signaling pathways, including WNT, NOTCH, BMP (bone morphogenetic protein), and FGF (fibroblast growth factor), play critical regulatory roles in the secondary heart field and contribute to the formation of the outflow tract and right ventricle [9]. Distinct changes in gene expression in these signaling pathways have been found in human heart tissues from individuals with TOF [10]. Haploinsufficiency of cardiac transcription factor genes that regulate these pathways (*NKX2.5, TBX1, TBX5* and *GATA4*) or the transmembrane receptors, *NOTCH1* and *NOTCH2* and their ligand *JAG1*, can cause TOF and other heart defects [8]. In addition, recent genome wide association studies (GWAS) of single nucleotide polymorphisms in subjects with TOF revealed a significant associations between SNPs located on chromosomes 12q24.11–13 and 13q32 [11], and 15q26.3 and 18q21.2 [12]. Additionally, somatic mutations that reduce the expression of *GATA6* have been associated with TOF [13].

Most non-syndromic CHDs, which account for 80% of all CHDs, occur sporadically, and families with clear monogenic inheritance of non-syndromic CHD are rare. This makes the identification of human disease genes involved in non-syndromic CHD by a classical positional genetics approach difficult [8]. There has been great interest in using aCGH to identify copy number variants (CNVs) that may contribute to CHD [14]–[22]. GWAS studies and aCGH have identified some new candidate risk factors for CHD, but there remains a significant amount of heritability that remains unexplained.

Genome-wide microarray-based comparative genomic hybridization (aCGH) or SNP microarray analysis is now recommended as a first-tier test for patients with intellectual disabilities, autism, and/or congenital anomalies [23], [24]. Considering that next generation sequencing (NGS) methods are not yet reliable enough to detect genomic CNVs; aCGH still represents the gold standard for identifying pathogenic chromosomal abnormalities as small as several hundred base pairs, and up to several megabases. We report our analysis of genomic DNA from 34 infants with sporadic non-syndromic TOF using a novel ultra high-resolution custom gene-centric comparative genomic hybridization (gcCGH) microarray. This array was used to identify CNVs involving genes known to be important for vertebrate heart development and/or function.

## Materials and Methods

### Ethics Statement

The Children’s Mercy Hospitals and Clinics institutional review board approved this research protocol. The parents of all subjects provided written consent for participation after reading the consent document and having their questions answered.

### Sample Collection

Genomic DNA was extracted from the peripheral blood of 34 infants with sporadic TOF (n _male_ = 20; n _female_ = 14; age range of 2 to 10 months). None of these infants had a 22q11.2 deletion, and most had no other clinical features (Clinical summary, Table S1). Gene expression analysis was done on right ventricular (RV) tissue that was discarded during reconstructive surgery, de-identified, flash frozen and stored in liquid nitrogen until processing. All tissue samples removed during surgery were excised by the performing surgeon for clinical indications utilizing standard of care procedures. The normally developing comparison RV tissues (cryo-preserved pulmonary homografts, described in detail in [25]) were thawed per protocol in sterile conditions. The attending surgeon (JEO) dissected the normally developing graft tissue to ensure the graft tissues were from the equivalent location as the tissue obtained from surgery.

### Construction of a Gene Centric Comparative Genomic Hybridization (gcCGH) Microarray

We used the Ingenuity Pathways Analysis (IPA, Redwood City, CA) database to derive a list of genes which contained the word “cardiac” or “heart” in their description (2237 IPA genes). In addition, we derived a refined subset of genes with validated roles in cardiovascular development or function (594 validated genes) using the three online databases: Cardiovascular Gene Ontology Annotation Initiative (http://www.ebi.ac.uk/GOA/CVI/prioritized_gene_list.html); CHD Wiki (http://homes.esat.kuleuven.be/~bioiuser/chdwiki/index.php/Main_Page); and HuGE Navigator (version 2.0) (http://www.hugenavigator.net/HuGENavigator/startPagePhenoPedia.do). Finally, in order to focus on genes that are more likely to impact cardiac development, we used IPA to derive a list of 229 genes that comprise five networks with experimentally confirmed roles in regulating vertebrate heart development: WNT; Notch; Sonic Hedgehog; cardiomyocyte differentiation via BMP receptors; and factors promoting cardiogenesis in vertebrates (development of these lists of genes is described in greater detail in [25] and the complete list is in Table S2).

The ultra high-resolution gene centric microarray was designed by Oxford Gene technology (OGT, Begbroke, Oxfordshire, UK). The genes from the five heart development networks and the 594 genes in our validated list were saturated with probes throughout the length of the gene, provided the sequence met length, GC content and Tm requirements for the array. The remaining genes from the IPA search had less dense probe assignment, and the remaining genome was covered at approximately 1 probe per 9.4 Kb.

### Array Hybridization

All gcCGH tests were performed, analyzed and validated based on the protocols described previously [26], [27]. In brief, all DNA samples were assessed for genomic DNA concentration and purity. Agarose gel electrophoresis was used to assess the quality of the genomic DNA samples. The test DNA (3 µg) and reference DNA (3 µg, pooled normal human male or female DNAs were used as reference DNA; purchased from Promega, Madison, WI) were digested with AluI and RsaI (Promega), and then labeled using the Agilent Genomic DNA Labeling Kit PLUS (Agilent Technologies, Santa Clara, CA). The individually labeled test and reference samples were then purified using Microcon YM-30 filters (Millipore, Billerica, MA). Following purification, the appropriately labeled test DNA and reference DNA were mixed together and hybridized to the custom gcCGH array. The hybridization was followed by four washing steps, and slides were scanned on an Agilent Microarray Scanner G2565BA with 5-µm resolution. Captured images were assessed with Feature Extraction Software, version 9.5 (Agilent), and the data were then imported into Agilent CGH Analytics 3.2.5 software for statistical analysis.

### aCGH Analysis

The analysis and visualization of aCGH-244 K data was performed using the Aberration Detection Method 2 (ADM2) within Agilent CGH Analytics 3.2.5 software as used in our clinical laboratory evaluations, and as described in detail previously [26]. Briefly, the ADM2 quality-weighted interval score algorithm identifies aberrant intervals in samples that have consistently high or low log ratios based on their statistical score. The score represents the deviation of the weighted average of the normalized log ratios from its expected value of zero calculated with Derivative Log2 Ratio Standard Deviation algorithm. Our threshold settings for the CGH analytics software to make a positive call were 6.0 for sensitivity, 0.25 for minimum absolute average log ratio per region, 10,000 for maximum number of aberrant regions, and 3 consecutive probes with the same polarity were required for the minimum number of probes per region.

### Exon-level Expression Microarrays

We previously analyzed the gene expression patterns in 13 of the subjects included in this genomic assessment. Detailed descriptions of subjects used for expression analysis and array preparation can be found in our previous publication [25]. Briefly, RNA was extracted from ∼10 mg of frozen right ventricular tissue using a mirVana microRNA isolation kit (Applied Biosystems/Ambion, Austin, TX) according to the manufactures instructions.

The exon-level expression arrays were Affymetrix HuEx-1_0-st-v2 (Affymetrix, Santa Clara, CA), and the experimental data were deposited in the Gene Expression Omnibus (GEO accession # GSE35776). These file are embargoed until June 2014, however, the files will be made available upon request prior to release from embargo. All arrays were run at the Kansas University Medical Center-Microarray Facility (KUMC-MF) according to the manufacturer’s protocols. The KUMC-MF is supported by the Kansas University-School of Medicine, KUMC Biotechnology Support Facility, the Smith Intellectual and Developmental Disabilities Research Center (HD02528), and the Kansas IDeA Network of Biomedical Research Excellence (RR016475). All analyses were performed using statistical software: Partek Genomics Suite software version 6.6 (Partek Inc, St. Louis). Raw data (CEL. files) were uploaded into Partek Genomics Suite for normalization and statistical analysis. Robust Multichip Analysis (RMA) was used for background correction followed by quantile normalization with baseline transformation to the median of the control samples. Only probes with intensity values above 20% of background value, in at least one of the conditions, were included for additional analysis. The Ingenuity Pathways Analysis (IPA) version 9.0 was used to explore networks, canonical pathways and predefined functional categories. A Fisher’s exact test was used to calculate a *p*-value determining the probability that the association between the genes in the dataset and the pathway was explained by chance alone. All biological functions and/or diseases in IPA’s database were considered for the analysis without bias. Significance was defined as a *p*-value ≤0.05.

### Real-Time Quantitative Polymerase Chain Reaction (q-PCR) and Quantitative Reverse Transcription-PCR (qRT-PCR)

CNV status was validated by q-PCR performed on a subset of genes/regions with copy number changes using specific primers (genes and primers are listed in Table S3 in Tables S3 and S4) and SYBR green as previously described [10], [28]. Gene expression was verified by qRT-PCR performed on a subset of genes using gene specific primers (primers are listed in Table S3) and SYBR green as previously described [10], [28]. For each sample, real-time q-PCR or qRT-PCR was performed in triplicate on an ABI 7000 sequence detection instrument. The point at which the intensity level crossed the PCR cycle threshold (C_T_) was used to compare individual reactions. We used *RNU24* and *GAPDH* to normalize our q-PCR or qRT-PCR results as done previously [10], [25], [27].

### UCSC Visualization

CNV and expression data were uploaded into the UCSC Genomics Bioinformatics browser website (http://genome.ucsc.edu/) in bed graph format according to the website instructions to allow for graphic inspection of the data. Expression level was based on comparison to tissue from normally developing individuals as described previously [10], [25].

## Results and Discussion

Heart formation and function are precisely controlled by networks of transcription factors that link upstream signaling systems with the protein-coding genes required for cardiac myogenesis, morphogenesis and contractility. Single gene defects have been identified which cause CHDs, but they are rare. Thus, most of the heritability of congenital heart defects remains to be identified. Several reports examining CNVs associated with CHDs to date have added several additional potential candidate genes/regions that may contribute to dysregulated heart development, but the resolution of these reports was limited to CNVs greater than about 100 kb. Therefore, we created an ultra high-density custom microarray focused on the genes known to be associated with cardiovascular development and function. We placed genes in three levels of importance based on potential impact on heart development using current annotation regarding potential developmental significance for the heart.

Our primary objective was to search for CNVs that were unlikely to be detected by previous assessments using standard aCGH or GWAS. We found many small CNVs that have the potential to impact the function of genes in our high priority list of genes, as well as the much larger number in the second and third tier genes. CNVs were detected for every chromosome within our cohort of children with TOF. Using the criteria described in the Methods section to identify CNVs, a total of 613 CNVs ranging from 74 base pairs to 19.5 Mb were detected. These included 402 deletions with an average size of 511 kb, and 211 duplications with an average size of 142 kb. We detected four CNVs larger than 5 Mb in size, and 216 that were less than 10 Kb. Summary information about the distribution and size of CNVs is listed in Table 1, and the specific distribution of these CNVs on individual chromosomes is listed in Table S4 in Tables S3 and S4. The entire list of CNVs is presented in Table S5 (the original aCGH files are available upon request from the first corresponding author).

**Table 1:** Summary of CNVs Detected in 34 Subjects using ultra-High Resolution CGH.

	Duplicated CNVs	Deleted CNVs	Total CNVs
Overall			
No. of CNVs	211	402	613
Range of CNVs (bp)	79–1,482,399	74–19,485,749	74–19,485,749
CNVs per individual	6.2	11.8	18.0
Total size (bp)	30,013,063	205,345,525	235,358,588
Average size per CNV (bp)	142,377	511,828	384,573
Average size per individual (bp)	1,158,165	1,423,255	2,581,420
No. of CNVs≥5 Mb (bp)	0	4	4

Among the CNVs detected in these 34 subjects, there were 16 subjects with 33 different CNVs involving 13 genes that are known to be critical for regulating development of the right ventricle making them good candidate genes for contributing to TOF (the genes were identified using the GO terms *right ventricle morphogenesis* or *outflow track morphogenesis*; Table 2). Two of these genes are known to be pathogenic, *JAG1* [18], [29] and *NOTCH1* [29], on chromosomes 9 and 20 respectively. Additionally, there were 79 genes from the list of 591 validated genes (genes that were experimentally determined to have a role in vertebrate heart development or function extracted from IPA) that were partially or completely contained in a CNV. Interestingly, all 34 individuals examined had at least one CNV involving these 79 genes. See Table S5 for a complete list of all CNVs detected.

### Comparison to CNVs in Individuals without Heart Defects

Our custom gene centered array was not available for analysis of subjects without heart defects. Therefore, in order to study whether the CNVs we identified were present in individuals without heart defects, we downloaded the datasets from Mills et al, and Jiang et al [30], [31]. These datasets were used for assessments of small CNVs in 24 individuals from the Hapmap project [30], and 32 individuals with autism [31] using genome sequencing tools. The subjects in these two reports had no reported evidence of heart defects. We eliminated SNPs and small indels less than 100 bp from our comparison. We manually searched the datasets for CNVs involving the 13 genes from pathways regulating heart development. We searched for small CNVs anywhere in the gene, or 10 kb upstream or downstream, in order to include regions where the CNV would be most likely to affect gene expression or function. These comparison dataset searches were limited to CNVs less than 10 kb (Table 2).

The dataset from Jiang et al [31] contained far more CNVs than the Mills et al [30] dataset. This is undoubtedly due to technical differences in the way the CNVs were identified, but may also reflect the higher burden of CNVs in general, in individuals with developmental disorders. Only one CNV involving our 13 critical genes did not have counterparts in the two comparison datasets: *HEY1,* (Table 2). HEY1 is a member of the Notch pathway, and is a transcription factor involved in determining cell fate and boundary formation. There were 4 deletions and 3 duplications involving *Hey1* in our cohort. *Hey1* knockout mice had no apparent phenotype, but the combined knockout of *Hey1* and *Hey2* caused embryonic lethality due to inadequate vascular remodeling [32]. Thus, *Hey1/Hey2* are critical for cardiovascular development. Therefore, alteration of the dosage of *HEY1* in these infants may have contributed to the inadequate communication between the first and second heart field and thus resulted in TOF and merits further investigation.

### Previously Identified Candidate Regions

One patient had a deletion encompassing *JAG1,* and a second deletion encompassing *NOTCH1.* A second patient had a deletion encompassing *NOTCH1* (Table 2). The JAGGED genes (*JAG1* and *JAG2*) encode NOTCH ligands. *NOTCH1* is one of four NOTCH family receptors. NOTCH is an ancient cell-signaling system that regulates cell fate via local cell-cell interactions, and can initiate diverse and tissue-specific downstream effects such as cell fate specification, progenitor cell maintenance, cell proliferation, apoptosis and boundary formation [33]. *JAG1* mutations have been found in patients with Alagille syndrome which can include TOF, and also in patients with non-syndromic right-sided heart defects such as pulmonary stenosis and TOF [29], [34]–[36]. Likewise, *NOTCH1* mutations have been shown to cause bicuspid aortic valve (BAV), as well as TOF [8]. These large deletions would be detected by standard aCGH, and are probable contributors to the TOF of these two infants.

The interest in identifying risk factors for CHD has resulted in recent large GWAS studies reporting significant associations between SNPs located on chromosomes 12q24.11–13 and 13q32 [11], and 15q26.3 and 18q21.2 [12]. We did not see any of our CNVs within or near these regions, with the exception of 15q26.3. We observed one subject with a 126 kb duplication in this region (see Table S5). However, it was detected by our background probes, and only impacted 3 open reading frame sequences; not within a target gene. This is an interesting coincidence, but is of uncertain significance. Additionally, a recent case report of somatic mutations that reduce the expression of *GATA6* have been associated with TOF [13]. We observed two deletions involving part of *GATA6,* and this strengthens the link between *GATA6* and TOF.

### Inheritance

The CNVs containing *JAG1, TBX2, GATA6* or *NOTCH1*were particularly interesting because of their known association with heart defects. Therefore, we tested these to determine whether they were de novo or inherited. Each CNV had at least one case that was inherited, and all but the CNV containing *JAG1* had a case that was de novo in origin (Table 2). Regardless of the origin of the CNV, CNVs involving some or all of a gene could make subtle differences in expression or function that could have contributed to TOF, and certainly warrant further investigation.

### CNVs in High Frequency in the TOF Cohort

Three CNVs containing genes associated with cardiovascular development or function, but not known to directly regulate heart development, occurred in high frequency in our cohort of infants with TOF: *CHL1, NKX2-1* and *GSTT1*. In addition to the above mentioned datasets, we used the Database of Genomic Variants (DGV, http://projects.tcag.ca/variation/) to search for additional reports of CNVs involving these three genes in normal individuals. These data are summarized in Table 2. Our cohort contained 19 CNVs (19 of 34; 56%) involving much of the promoter and the first 2 exons of *CHL1,* and all 19 CNVs were deletions. Two reports in the DGV indicated frequencies of 1 duplication in 30 subjects [37], and 2 duplications in 450 subjects [38]. In our cohort there were 13 deletions involving part of *NKX2-1* (13/34; 38%). The DGV contained one report with 2 deletions in 450 control subjects examined [38]. Finally, our cohort had 25 CNVs involving *GSTT1* (3 deletions and 22 duplications, 26/34; 76%). The frequencies reported in the DGV were 21 deletions in 30 subjects [37], and 630 CNVs (301 deletions and 329 duplications) in 1184 subjects [39]. The frequency of CNVs for these three genes in our cohort were all significantly higher than those seen in the DGV (chi square test of proportions, p<0.001) with the exception of the CNVs involving *GSTT1* compared to the observed frequency reported by Perry et al [37] (our TOF cohort, 26/34 = 76%; Perry et al. 21/30 = 70%), which, although there was a higher frequency in our cohort, it was not statistically significant.

Although CNVs involving these genes are relatively common in normal individuals, they occurred at a significantly higher frequency in our cohort of subjects with TOF. These three genes have no known direct influence on heart development. However, within the context of accumulating “noise” in the communication between the first and second heart field, alterations in the behavior of these genes might contribute to the dysregulation that leads to TOF.

### Correlation between CNVs and Gene Expression

We had genome wide expression exon arrays available for 13 of our subjects who also underwent gcCGH analysis. The expression analysis was done using RNA extracted from right ventricular tissue removed at the time of surgical correction of the defect [10], [25]. We examined the data from these two platforms to look for correlations between CNVs and gene expression in the RV. We used gene expression data at the probe (exon) level, as well as the whole gene level, to test for CNV/expression correlation. We limited our correlational assessment to CNVs associated with the genes in the list of 591 validated genes. Each of the 13 subjects with expression analysis had at least one CNV involving one or more genes on the list. In these 13 subjects, there were a total of 79 CNVs meeting the inclusion criteria. There were 41 different genes with a CNV encompassing part or all of the genes, never the less, most of the genes within the CNVs occurred too infrequently to allow conclusions to be made about the correlation between the CNV and expression in the ventricular tissue.

**Figure 1:**
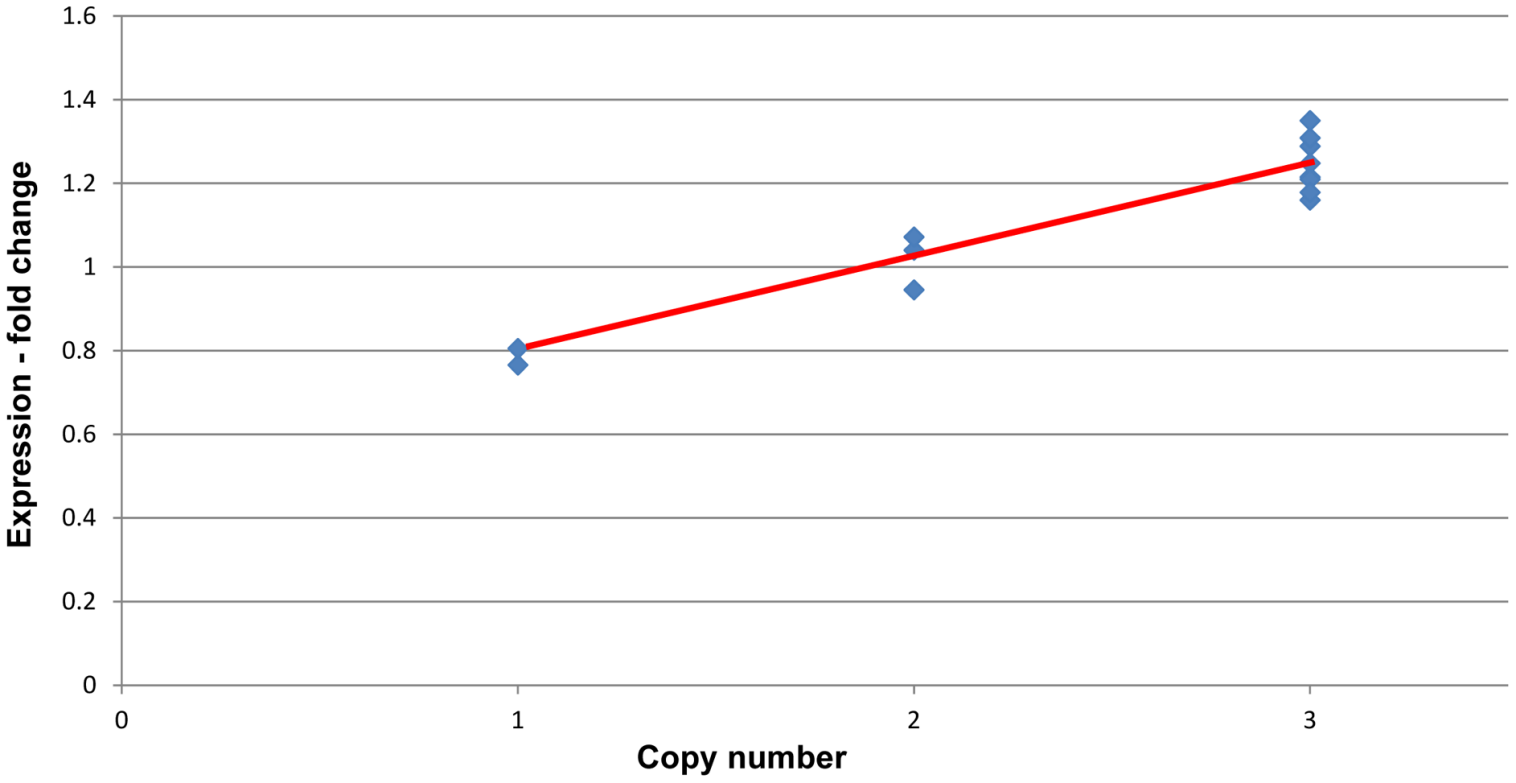
Correlation between copy number and gene expression of *GSTT1*. *GSTT1* expression in tissue from the right ventricle of infants with TOF relative to controls. Pearson correlation coefficient; r = 0.95, p = 0.001.

*GSTT1* was the only gene with a clear correlation between copy number and gene expression in the RV (Pearson’s correlation coefficient, r = 0.95, p = 0.001; 7 duplications, 2 deletions from the 13 subjects examined with expression arrays). Figure 1 shows the relationship between *GSTT1* copy number and gene expression in the RV tissue of infants with TOF (compared to RV control tissue). We uploaded the CGH data and the probe intensity data to the UCSC browser to visualize the relationship between the CNVs and probe level expression (Figure S1). The TOF subjects without CNVs involving *GSTT1* did not have significant changes in *GSTT1* expression when compared to the tissue from normally developing controls. Those with deletions had significantly less *GSTT1* expression, and those with duplications had significantly more expression of *GSTT1* in the right ventricular tissue, compared to normally developing subjects. This included all the probes within the affected region (Figure S1). *GSTT1* is the first step in glutathione detoxification, and null genotypes of glutathione transferases have recently been associated with risk of CHD, particularly when parents were exposed to environmental toxins [40]. But since our cohort with TOF had individuals with under- and over-expression of *GSTT1* relative to control subjects, it seems rather unlikely that CNVs involving *GSTT1* represent a strong risk factor for TOF.

In addition, we used qRT-PCR to examine the expression of 5 genes of particular relevance for heart development, *TBX2* (3 duplications, 1 deletion), *GATA6* (2 deletions), *NOTCH1* (2 deletions), *NOTCH2* (2 deletions) and *JAG1* (1 deletion) in RV tissue from subjects with CNVs involving these genes compared to the pooled RNA from our control samples (data not shown). We did not have exon expression microarrays on any of these subjects, but extracted RNA from archived RV in order to examine expression. Expression of *NOTCH1, GATA6* and *JAG1* did not significantly differ from the expression of the control samples regardless of CNV status. Interestingly, the expression of *TBX2* and *NOTCH2* were reduced in the RV tissue of all TOF subjects for whom we examined expression data relative to the control samples from normally developing infants. This no doubt reflects other regulatory mechanisms that are independent of copy number.

It should be emphasized that our expression data was limited in scope to a small sample, and expression at 6 months of age may not reflect expression during early gestation when the heart fields were misaligned. In addition, subtle changes in expression that are not within the resolution of our tools could be important for precise regulation of early heart development.

## Conclusions

Our study was designed to examine structural variation at very high resolution in genes with potential significance for heart development or function. We identified a large number of relatively small CNVs, not previously reported. With the possible exception of the CNVs involving *JAG1* and *NOTCH1*, our small sample size precludes any conclusion about the broader impact of these CNVs. However, it seems likely that some of these CNVs, together with other genetic and epigenetic variants, and probably environmental factors, may have contributed to the developmental deficiency that caused TOF. Delineating the impact of multiple small contributions to developmental irregularity remains a challenge. However, new high-resolution technologies are aiding in the identification of potentially important genetic elements that may eventually provide a molecular genetic explanation for complex developmental disorders like tetralogy of Fallot.

## Supporting Information

Figure S1 **Representative examples of copy number variants involving GSTT1 and expression of GSTT1 in cardiac tissues from infants with TOF relative to controls as graphically displayed in the UCSC Genome Browser.**
(TIF)

Table S1 **Patient Characteristics.**
(XLSX)

Table S2 **List of genes used to create the gene centric array.**
(XLSX)

Tables S3 and S4 **Includes Table S3 and S4.** Table S3. Primers used for CNVs containing genes of primary importance for heart development. Table S4. Summary of chromosomal distribution of CNVs.
(PDF)

Table S5 **Complete list of CNVs.**
(XLS)
