# Correction: Ultra High-Resolution Gene Centric Genomic Structural Analysis of a Non-Syndromic Congenital Heart Defect, Tetralogy of Fallot

**DOI:** 10.1371/journal.pone.0099148

**Published:** 2014-05-27

**Authors:** The PLOS ONE Staff

Table 2 is missing in the XML and PDF versions of the article. Please see Table 2 below.

**Table 2:** CNVs containing genes with a known association with right ventricular development^1^

Chromosome	Gene	Band	Start^2^	Stop^2^	Size^2^	# in TOF cohort / total in cohort	inheritance	Mills et al 2006^3^ # with CNV / total in cohort	Jiang et al 2013^3^ # With CNV / total in cohort	DGV # With CNV/ total in cohort
chr20	JAG1	p12.2	10,653,282	10,654,729	1,447	1del/34	from mother	0 / 24	2 /32	
chr17	TBX2	q23.2	59,477,025	59,479,140	2,115	3dup,1del/34	3 denovo / 1 dup from father	0 / 24	3/ 32	
chr18	GATA6	q11.2	19,749,386	19,761,617	12,231	2del/34	1 denovo / 1 from mother	0 / 24	9 / 32	
chr9	NOTCH1,	q33.3 - q34.3	130,158,557	140,785,695	10,267,138	2del/34	1 denovo / 1 from mother	10 / 24	7 / 32	
chr8	HEY1	q21.33	8,0679,052	80,727,908	48,856	3dup, 4del/34		0 /24*	0 /32**	
chr1	RYR2	q43	237,813,301	237,817,763	4,462	2del/34		2/24	13 /32	
chr8	FOXH1	q24.3	142,286,322	145,755,059	3,468,737	2del/34		0 /24	1 /32	
chr1	NOTCH2	p12 - p11.2	120,529,652	120,612,294	82,642	2del/34		1 / 24	0 / 32	
chr16	SOX8	p13.3	126,558	3,790,511	3,663,953	2del/34		2 / 24	4/ 32	
chr3	DVL3	q27.1	183,872,674	184,242,147	369,473	2del/34		0 / 24	4/32	
chr6	SOX4	p22.3	21,594,198	21,596,935	2,737	1del/34		0 / 24	1/32	
chr7	LFNG	p22.3	165,353	2,706,165	2,540,812	1del/34		0 / 24	9/ 32	
chr8	CHD7	q12.1	61,582,823	61,591,393	8570	1dup,2del/34		0 / 24	2/32	
chr14	NKX2.1^4^	q13.3	36,986,373	36,991,296	4,923	12/34 all deletions		0 / 24***	2 / 32***	2/450***
chr3	CHL1^4^	p26.3	115,283	305,668	190,385	19/34 all deletions		1 / 24***	2/32***	1/30[37]*** and 2/450[38]***
chr22	GSTT1^4^	q11.23	24,236,629	24,384,403	147,774	3 del,22 dup/34		0 / 24***	2/ 32***	21/30[37] and 630/1184[39]***
										

1 the genes were identified using the GO terms *right ventricle morphogenesis or outflow track morphogenesis*

2 If there is more than one CNV, start, stop and size represent the largest CNV

3 Mills et al. [30] and Jiang et al.[31] datasets were downloaded and searched for small CNVs (> 100 bp to 10kb) anywhere in the gene or in the 10kb upstream or downstream to include the promoter and 3’ end.

4 NKX2.1, CHL1 and GSTT1 do not have a direct connection to RV development but were included because of the high frequency of CNVs involving them in our cohort

*^,^ **^,^ ***significant difference p<0.05, 0.01, 0.001
