# Supplementary figures and images for: Ultra High-Resolution Gene Centric Genomic Structural Analysis of a Non-Syndromic Congenital Heart Defect, Tetralogy of Fallot
Source: PLoS One. 2014 Jan 31;9(1):e87472. doi: 10.1371/journal.pone.0087472 (PMC3909147; doi:10.1371/journal.pone.0087472)

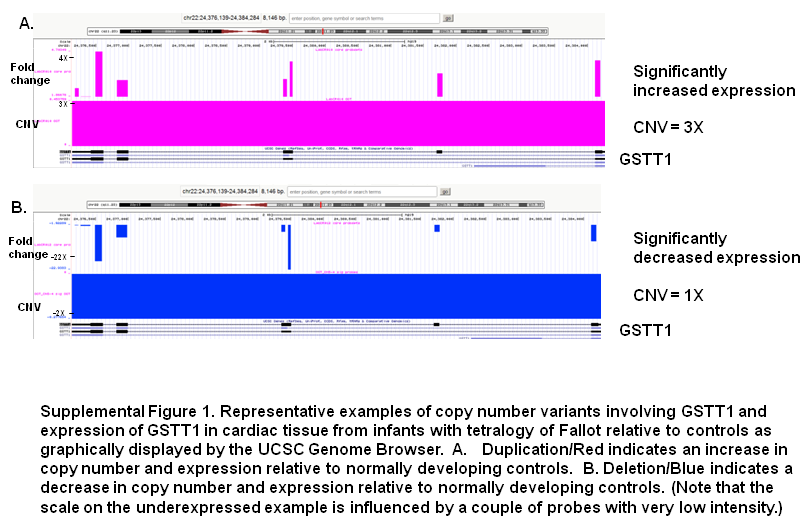

Supplement: Figure S1 — Representative examples of copy number variants involving GSTT1 and expression of GSTT1 in cardiac tissues from infants with TOF relative to controls as graphically displayed in the UCSC Genome Browser. (TIF) [file pone.0087472.s001.tif]
