# Supplementary material for: Ultra High-Resolution Gene Centric Genomic Structural Analysis of a Non-Syndromic Congenital Heart Defect, Tetralogy of Fallot
Source: PLoS One. 2014 Jan 31;9(1):e87472. doi: 10.1371/journal.pone.0087472 (PMC3909147; doi:10.1371/journal.pone.0087472)
Supplement: Tables S3 and S4 — Includes Table S3 and S4. Table S3. Primers used for CNVs containing genes of primary importance for heart development. Table S4. Summary of chromosomal distribution of CNVs. (PDF) [file pone.0087472.s004.pdf]

**Supplemental Table 3. Primers for CNVs containing genes of primary importance to heart development**				
**Name**	**Chromosome**	**Fragment Size**	**Forward**	**Reverse**
JAG1	Chr20	125bp	CTCCTTGAGGCATTTGA	TGTCCATGCAGAACGTGAA
GATA6	CHr18	145bp	ATCTCTTCCTCGTCCTC	GATGCGAAGCGTAGGAACT
NOTCH1	Chr9	142bp	AACGAGGTCGGCTCCTA	CACACTCGTGGGTGACG
TBX2	Chr17	135bp	CTGTAGCCCAGTTCCCAATAC	GCGGCTACAATCTCCATCA
NKX2-1	Chr14	101bp	TGGTCTACGTGTCTGTCAGTCTGT	CGCTCGGATTCTCTCCGGTA
OFD1 (control for normalization)	ChrX	137bp	AGGTGTTCTGCTGCTGAGATGGAA	TCCCTTTGTGCCCAGATGAAGAGA

Supplemental Table 4. Summary of Chromosomal Distribution of CNVs

Chromosome	Duplicated CNVs	Deleted CNVs	Total CNVs
1	1	24	25
2	2	21	23
3	11	32	43
4	26	15	41
5	41	24	65
6	14	44	58
7	4	14	18
8	12	30	42
9	1	12	13
10	10	9	19
11	2	9	11
12	5	5	10
13	3	6	9
14	7	34	41
15	2	32	34
16	8	11	19
17	22	39	61
18	0	6	6
19	8	3	11
20	0	8	8
21	0	7	7
22	27	7	34
X	5	10	15

bp, base pairs; CGH, comparative genomic hybridization; CNVs, copy number variants.
